# Supplementary material for: Multimodal vaccination targeting the receptor binding domains of Clostridioides difficile toxins A and B with an attenuated Salmonella Typhimurium vector (YS1646) protects mice from lethal challenge
Source: Microbiol Spectr. 2024 Jan 8;12(2):e03109-22. doi: 10.1128/spectrum.03109-22 (PMC10846063; doi:10.1128/spectrum.03109-22)
Supplement: Supplemental material — Tables S1 and S2; Figures S1, S2, and S3. [file spectrum.03109-22-s0004.pdf]

Supplemental Table 1 – Primers used in this study for Tn7 plasmid construction

	Forward Primer (5' --> 3')	Reverse Primer (5' --> 3')	Source
pagC_SspH1_rbdA	CCCGGGCTGCAGGAATTCACGAGGCCCTTTCGTCTTCA	AGGCCTTCGCGAGGTACCGCTTGGCTGCAGATCTTTAACCG	(26)
SspH2_SspH2_rbdA	CCCGGGCTGCAGGAATTCACGAGGCCCTTTCGTCTTCA	AGGCCTTCGCGAGGTACCGCTTGGCTGCAGATCTTTAACCG	(26)
frr	CCCGGGCTGCAGGAATTCCTGCTGCGTAATAACCGTGT	GGATATTAAACATGTTACGAATCCTTGAAAACT	YS1646 genome
SspH1_rbdA	AGGATTCGTAACATGTTTAATATCCGCAATACACAACCTTCT	AGGCCTTCGCGAGGTACCGCTTGGCTGCAGATCTTTAACCG	(26)
pagC_SspH1_rbdB	CCCGGGCTGCAGGAATTCACGAGGCCCTTTCGTCTTCA	AGGCCTTCGCGAGGTACCGCTTGGCTGCAGATCTTTAACCG	(26)
SspH2_SspH2_rbdB	CCCGGGCTGCAGGAATTCACGAGGCCCTTTCGTCTTCA	AGGCCTTCGCGAGGTACCGCTTGGCTGCAGATCTTTAACCG	(26)
frr	CCCGGGCTGCAGGAATTCCTGCTGCGTAATAACCGTGT	AGACAGCATCATGTTACGAATCCTTGAAAACT	YS1646 genome
SspH2_rbdB	AGGATTCGTAACATGATGCTGTCTGGTCAGCG	AGGCCTTCGCGAGGTACCGCTTGGCTGCAGATCTTTAACCG	(26)

Supplemental Table 2 – Symptom Scoring Chart for *C. difficile* Infection

	Scores				
**Category**	**0**	**1**	**2**	**3**	**4**
**Weight Loss**	None	<10%	10<15%	15<20%	≥20%
**Activity**	Normal	Alert/slow moving	Lethargic/shaky	Inactive unless prodded	Not moving
**Posture**	Normal	Back slanted	Hunched	Hunched/nose down	
**Coat**	Normal	Piloerection	Rough Skin	Very ruffled/puff/ungroomed	
**Diarrhea**	Normal	Soft Stool/discolored (yellowish)	Wet stained tail/mucous +/- blood	Liquid/no stool (ileus)	
**Eyes/Nose**	Normal	Squinted 1/2 closed	Squinted/Discharge	Closed/discharge	

Euthanise at Clinical Score ≥14 and/or Weight loss score of 4

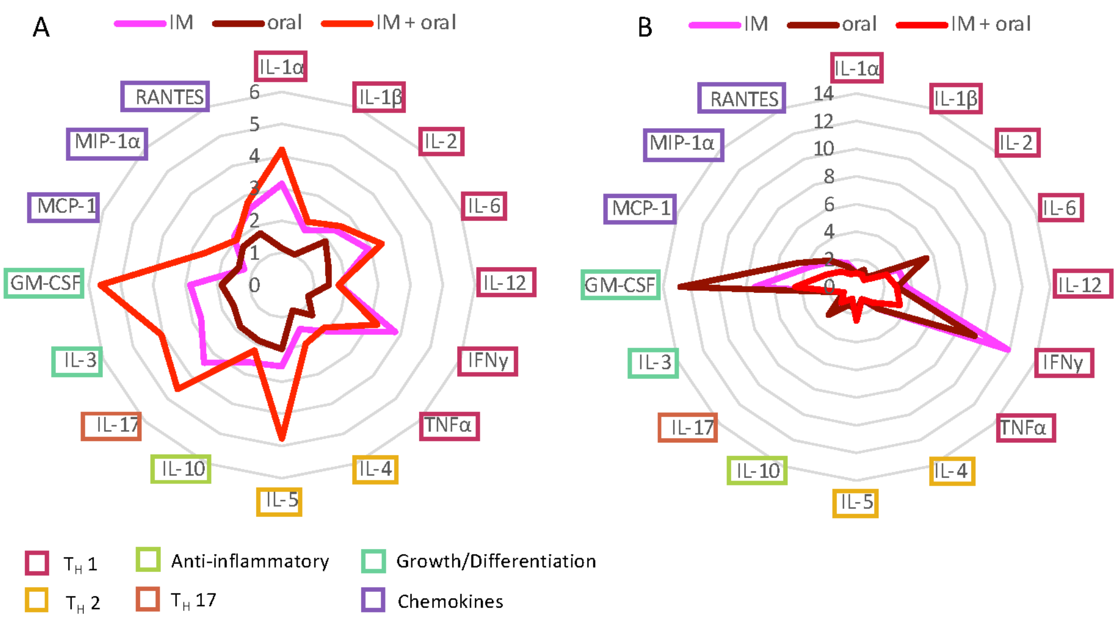

Supp Fig. 1: Vaccination elicits increased cytokine expression in the mesenteric lymph nodes and Peyer's patches

Mice were vaccinated with 3 µg of recombinant antigen (rrbdA) intramuscularly (IM) on day 0, with 3 doses of $1x10^8$ cfu of YS1646 given orally (PO) on days 0, 2 and 4. 32 days after vaccination, the mesenteric lymph nodes (mLN) and Peyer's patches (PP) were collected, and cells were isolated. Cells were stimulated for 72h with rrbdA and the supernatant was collected and examined by ELISA to evaluated cytokine and chemokine secretion. (n=6, 2 repeats) **A** Cytokine and chemokine secretion from mLN cells is shown. **B** Cytokine and chemokine secretion from cells in the PPs is shown. All data are shown as the fold change of the mean of secreted cytokines and chemokines in experimental groups compared to the PBS control.

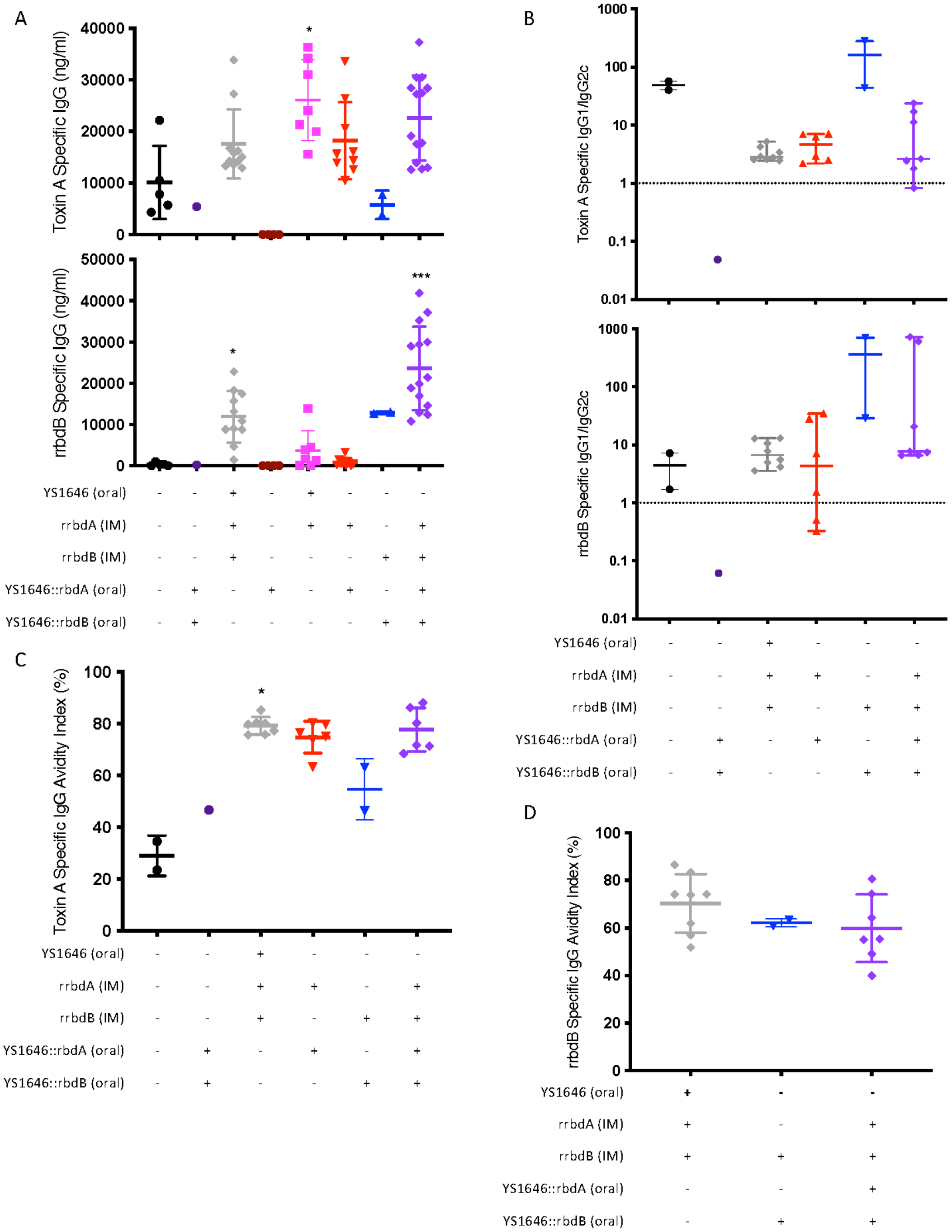

Supp Fig. 2: Vaccination elicits higher systemic IgG titers after challenge, compared to unvaccinated survivors.

Mice were vaccinated with 3 µg of recombinant antigen (rrbdA/rrbdB) intramuscularly (IM) on day 0, with 3 doses of $1x10^8$ cfu of YS1646 delivered orally (PO) on days 0, 2 and 4. At 5 weeks after vaccination, mice were challenged PO with 1370-2500 cfu of freshly cultured *C. difficile*. Mouse symptoms were scored 1-3 times daily by an observer blind to the treatment received. Mice that received a score of 14/20 or above or had over 20% weight loss from their starting weight were at the humane endpoint and were euthanized. Serum of surviving mice was collected 3 weeks after challenge and IgG titers were determined by ELISA. **A** Toxin A-specific

and rrbdB-specific IgG titers are shown as mean with standard deviation (SD) and a multiple comparison test to compare all groups, except for the surviving mouse vaccinated PO only against both TcdA and TcdB, to the PBS control group (n=1-14, 2 repeats). **B** Toxin A- and rrbdB-specific IgG1/IgG2c ratio was determined by (antigen specific IgG1 titers)/(antigen specific IgG2c titers). A titer below detection was set to 48.75 ng/ml, half of the level of detection. Data are shown as the median and 95% confidence intervals, with a multiple comparison test comparing all groups, except for the sole surviving mouse vaccinated PO only against both TcdA and TcdB, to the PBS control group (n=1-8, 1 repeat). **C** Toxin A-specific IgG avidity index was determined by (antigen-specific IgG concentration remaining after 6M urea incubation)/(total IgG concentration) x 100% . Data are shown as the mean and SD, with a multiple comparison test comparing all groups, except for the surviving mouse vaccinated PO only against both TcdA and TcdB, to the PBS control group (n=1-8, 1 repeat). **D** rrbdB-specific IgG avidity index was determined the same as in (**C**). Only groups with consistently high rrbdB-specific IgG titers were tested for avidity. Data are shown as the mean and SD, with a multiple comparison test comparing all groups to the rrbdA/B + YS1646 group (n=2-8, 1 repeat). All panels were analysed using the Kruskal-Wallis test and Dunn's multiple comparison test. *P* values without a bracket are in comparison to the PBS control group. *, $P < 0.05$; ***, $P < 0.001$.

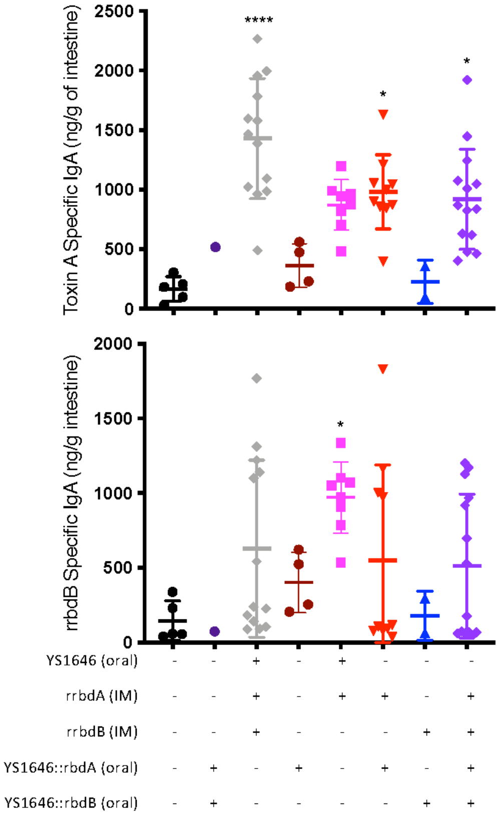

Supp. Fig. 3: Vaccination increases IgA titers in the intestine after challenge, compared to unvaccinated survivors.

A Mice were vaccinated with 3 µg of recombinant antigen (rrbdA/rrbdB) intramuscularly (IM) on day 0, with 3 doses of $1x10^8$ cfu of YS1646 delivered orally (PO) on days 0, 2 and 4. At 5 weeks after vaccination, mice were challenged with po delivered 1370-2500 cfu of freshly cultured *C. difficile*. Mouse symptoms were scored 1-3 times daily by an observer blind to the treatment received. Mice that received a score of 14/20 or above or had over 20% weight loss from their starting weight were at the humane endpoint and were euthanised. The small intestine of surviving mice was collected 3 weeks after challenge and IgA titers were determined by ELISA. Toxin A-specific and rrbdB-specific IgA titers are shown as mean with standard deviation (SD) and a multiple comparison test to compare all groups, except for the sole surviving mouse vaccinated PO only against both TcdA and TcdB, to the PBS control group (n=1-13, 2 repeats). All P values are in comparison to the PBS control group. *, $P < 0.05$; ****, $P < 0.0001$.
